# Notice

**Published:** 1855-03

**Authors:** 


### Notice.

### NEW YORK ACADEMY OF MEDICINE.

### PRIZE ESSAY.

THE undersigned, a committee appointed by the New York Academy of Medicine, hereby give notice that a prize of ONE HUNDRED DOLLARS will be awarded to the author of the best essay on the subject of *Cholera Infantum.*

Each communication must be accompanied by a sealed packet, containing the name of the author, which will be opened only in the case of the successful competitors. Unsuccessful communications will be returned, on application, after April 1st, 1856.

Communications must be addressed (post-paid) to the Chairman of the Committee, Dr. Joseph M. Smith, No 11 East 17th street, New York, on or before the 1st of January, 1856,

JOSEPH M. SMITH, M. D.
ISAAC WOOD, M. D.
H. D. BULKLEY, M. D.
JOHN W. CORSON, M. D.
S. S. PURULE, M. D.

NEW YORK, *February* 23, 1855.

